# The Relationship Between Digit Ratio (2D:4D) and Aspects of Cardiorespiratory Fitness: A Systematic Review and Meta‐Analysis

**DOI:** 10.1002/ajhb.70040

**Published:** 2025-04-04

**Authors:** Bethany Gower, Matthew Russell, Jordan M. Tomkinson, Samantha J. Peterson, Marilyn G. Klug, Grant R. Tomkinson

**Affiliations:** ^1^ Alliance for Research in Exercise, Nutrition and Activity (ARENA), Allied Health and Human Performance University of South Australia Adelaide South Australia Australia; ^2^ Department of Education, Health and Behavior Studies University of North Dakota Grand Forks North Dakota USA; ^3^ Allied Health and Human Performance University of South Australia Adelaide South Australia Australia; ^4^ School of Medicine & Health Sciences University of North Dakota Grand Forks North Dakota USA; ^5^ Department of Population Health University of North Dakota Grand Forks North Dakota USA

**Keywords:** cardiorespiratory fitness, cross‐sectional studies, digit ratios, oxygen consumption, physical fitness, testosterone

## Abstract

**Introduction:**

Digit ratio (2D:4D), a proxy of prenatal testosterone exposure, is a putative marker of physical fitness. No study has comprehensively synthesized studies examining associations between 2D:4D and cardiorespiratory fitness (CRF). We aimed to systematically review and meta‐analyze studies reporting associations between 2D:4D and aspects of CRF.

**Methods:**

We systematically searched the literature for full text, refereed, cross‐sectional studies reporting Pearson's correlation coefficients between objectively measured 2D:4D and at least one aspect of CRF. CRF was objectively assessed using field‐based measures (maximal long‐duration exercise performance) or laboratory‐based measures (maximal oxygen uptake [VO_2max_], ventilatory threshold [VT], or mechanical efficiency [ME]). We used random‐effects meta‐analysis to estimate the pooled correlation and 95% confidence interval (95% CI) for aspects of CRF, and moderator analyses to estimate the influence of sex and age.

**Results:**

Data from 22 studies, representing 5293 individuals (54% male; mean age range = 10.1–40.2 years) from 12 countries were included. We found a significant strong negative correlation for VT (*r* = −0.61, 95% CI = −0.78, −0.37) and a significant weak negative correlation for exercise performance (*r* = −0.18, 95% CI = −0.25, −0.10), indicating that individuals with lower 2D:4Ds had higher VT and better exercise performance. No significant correlations were found for VO_2max_ or ME. Neither sex nor age were significant moderators, except for age which moderated the correlation for exercise performance.

**Conclusions:**

2D:4D is a proxy for some aspects of CRF like exercise tolerance (i.e., VT) and performance, but not other aspects like aerobic capacity and efficiency.

## 1 Introduction

The ratio of the lengths of the second (2D, the index finger) and fourth (4D, the ring finger) digits in humans is known as the digit ratio (2D:4D). The 2D:4D appears set by the end of the first trimester of pregnancy (Galis et al. 2010; Malas et al. 2006) and is stable throughout life (McIntyre et al. 2005; Trivers et al. 2006). Males generally have lower 2D:4Ds than females (Bernardino et al. 2025; Manning et al. 1998), which may be due to sex differences in prenatal testosterone and estrogen levels, as there are proportionally more receptors for androgen and estrogen on 4D than 2D (Manning 2008; Zheng and Cohn 2011). While these are some of the reasons why 2D:4D is considered a retrospective marker of prenatal exposure to testosterone and estrogen (Manning and Fink 2023; Swift‐Gallant et al. 2020), controversy remains, and some hold an opposing view (McCormick and Carré 2020).

Prenatal testosterone exposure has long‐term (organizational) effects on the growth and development of several physiological systems (e.g., cardiovascular, musculoskeletal, central nervous) (Manning et al. 2014; Zheng and Cohn 2011), and may influence the short‐term (activational) effects of testosterone in later life by priming the endocrine system to respond to challenging situations (e.g., intense exercise) by suddenly spiking testosterone levels (Cook and Crewther 2012; Crewther et al. 2011, 2022). Theoretically, individuals with higher exposure to prenatal testosterone should have lower 2D:4D and higher physical fitness levels. Meta‐analyses have indicated that 2D:4D is significantly and negatively associated with sports and athletic performance as well as aspects of physical fitness (Hönekopp and Schuster 2010; Pasanen et al. 2022). In their meta‐analysis of 21 samples representing 2527 individuals, Hönekopp and Schuster (2010) reported that 2D:4D was a significant weak negative correlate of sports and athletic performance. Hönekopp and Schuster (2010) pooled data across various objective and self‐ or coach‐reported measures, including maximal long‐duration exercise performance requiring high cardiorespiratory fitness (CRF) (e.g., long‐distance running/walking) and maximal short‐duration exercise performance requiring high muscular fitness (e.g., handgrip strength, short‐distance sprinting), by comparing athletes to the general population or athletes competing at different standards. This may have contributed to their finding of substantial between‐study heterogeneity. In a more focused review, Pasanen et al. (2022) meta‐analyzed 22 studies representing 5271 individuals and reported associations between 2D:4D and objectively assessed muscular fitness (measured as handgrip strength), also finding a significant weak negative correlation. However, there has never been a comprehensive study that has systematically synthesized the literature and meta‐analyzed studies that have quantified the association between 2D:4D and objectively assessed aspects of CRF.

CRF reflects the capacity of the physiological systems to perform whole‐body (or large muscle), dynamic, physical activity for long periods (American College of Sports Medicine 2021). CRF, an important measure of health as well as sports and athletic performance (Armstrong et al. 2011; Lang et al. 2024), can be objectively assessed in laboratory or field settings. Maximal oxygen uptake ($\dot{V}O_{2\max}$), the highest rate of oxygen consumption during maximal aerobic exercise, is considered the criterion measure of CRF (Ross et al. 2016). $\dot{V}O_{2\max}$ is best measured in the laboratory using indirect calorimetry. However, $\dot{V}O_{2\max}$ does not describe all aspects of CRF. Other aspects of CRF include lactate or ventilatory threshold (i.e., the exercise intensity at which there is a rapid increase in blood lactate levels or ventilation relative to the rate of oxygen consumption) and mechanical efficiency (i.e., the oxygen cost for any given exercise intensity) (Léger 1996). When these laboratory assessments are not feasible, CRF can be assessed in the field using validated maximal aerobic exercise performance tests involving long‐distance running/walking or shuttle running. In the past decade, numerous studies have examined the association between 2D:4D and various objectively measured aspects of CRF (especially field‐based measures) across different demographic groups (e.g., Hull et al. 2015; Parpa et al. 2024; Ranson et al. 2015). This preponderance of data provides a timely opportunity for a focused systematic review of the published literature to better understand such relationships. The aim of this study, therefore, was to systematically review and meta‐analyze studies reporting associations between 2D:4D and objectively assessed aspects of CRF. This research may improve our understanding of the biological basis of CRF, have implications for health as well as sports and athletic performance, and provide important insights for future research.

## 2 Materials and Methods

### 2.1 Protocol and Registration

This systematic review and meta‐analysis protocol was prospectively registered with Open Science Framework (registration number: https://doi.org/10.17605/OSF.IO/C36WE) on the 21 December 2023. Reporting followed the Reporting Items for Systematic reviews and Meta‐Analyses (PRISMA) statement (Page et al. 2021).

### 2.2 Eligibility Criteria

Studies were included if they met the following criteria:

1. *Population*: Children (aged < 18 years) and/or adults (aged ≥ 18 years). Studies specifically on participants with known disease, illness, or injury were excluded.
2. *Measures*: Objectively assessed (a) 2D and 4D lengths (e.g., manually using bone calipers, electronically using Cartesian coordinate geometry) and (b) aspects of CRF including field‐based measures (i.e., maximal aerobic exercise performance [e.g., long‐duration exercise performance or estimated $\dot{V}O_{2\max}$ derived from maximal exercise work rate]) and laboratory‐based measures (e.g., $\dot{V}O_{2\max}$ or peak oxygen uptake [$\dot{V}O_{2\text{peak}}$] [herein termed $\dot{V}O_{2\max}$ for simplicity] in mL/kg/min or metabolic equivalents of task, or other aspects derived from graded exercise testing using indirect calorimetry, such as ventilatory threshold [VT; i.e., the second ventilatory threshold, also called the *respiratory compensation point*] or mechanical efficiency [ME]). Other measures/estimates of 2D and 4D lengths (e.g., self‐reported) or CRF (e.g., self‐ or coach‐reported personal best physical performances, race placings) were excluded.
3. *Outcome*: Bivariate correlation coefficients (Pearson's *r* or Spearman's *ρ*) between 2D:4D and CRF. As recommended by Aloe and Thompson (2013), partial effect sizes for correlation (e.g., partial correlations, standardized beta coefficients) were excluded.
4. *Study design*: Unique observational (cross‐sectional) studies. Other study designs (e.g., randomized controlled trial, experimental, case–control, cohort) were excluded.
5. *Publication status*: Full text, refereed, published journal articles. Other publication types (e.g., conference abstracts/papers, commentaries, editorials, dissertations) were excluded.

### 2.3 Information Sources

We identified studies by searching four online databases, one web search engine, and other sources (e.g., reference lists, topical reviews, personal libraries) as recommended by the PRISMA statement (Page et al. 2021). Database and web searches were performed in MEDLINE (via Ovid), SPORTDiscus (via EBSCOhost), Embase (via Ovid), Web of Science (Core Collection), and Google Scholar (first 200 results sorted by relevance) from inception to the 13 August 2024.

### 2.4 Search Strategy

We designed the database and web search strategy in consultation with an academic librarian experienced in systematic reviews. No date or language restrictions were imposed. The search strategy for databases is shown in Data S1.

### 2.5 Selection Process

Records were imported first into EndNote (Clarivate Analytics; Philadelphia, PA, USA), where they were de‐duplicated, and then into Covidence (Veritas Health Innovation, Melbourne, VIC, Australia) for further de‐duplication and record screening. Titles and abstracts and then full‐text articles were independently screened against inclusion criteria by two of the following authors (M.R., J.M.T., and G.R.T.). Conflicts were resolved by a third author not involved in the conflict (e.g., G.R.T. for conflicts between M.R. and J.M.T.).

### 2.6 Data Collection Process

Data were independently extracted by one of the following authors (M.R. or G.R.T.) using a pre‐designed Excel spreadsheet (Microsoft, Redmond, WA, USA). Another author (J.M.T.) checked the extracted data for accuracy. We requested additional information from corresponding authors of included studies when necessary.

### 2.7 Data Items

The following data were extracted from each study: author(s), country, publication year, participant demographics (e.g., sex/gender, age, ethnicity), sampling details (method, strategy), sample size, 2D:4D data (e.g., protocol, hand, means, standard deviations [SDs]), CRF (e.g., measure, protocol, means, SDs), bivariate correlation coefficients (e.g., *r*‐ or *ρ*‐values, *p*‐values, standard errors, 95% confidence intervals [95% CI]), and the full citation.

### 2.8 Study Risk of Bias Assessment

Risk of bias was assessed by the Joanna Briggs Institute (JBI) critical appraisal checklist for analytical cross‐sectional studies (Moola et al. 2017). This 8‐item checklist assesses the methodological quality and the potential for bias in design, conduct, and analysis. We defined each appraisal item prior to assessment. A score of “Yes,” “No,” “Unclear,” or “Not Applicable” was given for each appraisal item, with “Yes” and “Not Applicable” answers indicative of a lower risk of bias. The frequency of “Yes'” and “Not Applicable” scores were used to indicate the overall risk of bias for each study. Study risk of bias was independently assessed by two authors (B.G. and J.M.T.), with conflicts resolved by a third author (G.R.T.).

### 2.9 Effect Measures and Synthesis Methods

We used bivariate correlation coefficients as the measure of effect size. We calculated and included study‐sex‐specific mean correlations for unique participant groups (i.e., for each study and sex, the mean correlation was calculated for different age or physical activity groups) when multiple correlation coefficients were reported in a study. Negative correlations indicated that individuals with lower 2D:4Ds had higher CRF, while positive correlations indicated that individuals with lower 2D:4Ds had lower CRF. The magnitude of correlation was interpreted as negligible (*r* ≤ 0.10), weak (*r* = 0.10–0.29), moderate (*r* = 0.30–0.49), or strong (*r* ≥ 0.50) (Cohen 1988).

All analyses were conducted in IBM SPSS Statistics (v27, IBM, Chicago, IL, USA) and R (v4.3.0, R Foundation for Statistical Computing, Vienna, Austria) using syntax files provided by Field and Gillett (2010). A random‐effects model was used to pool correlations and 95% confidence intervals (CI), stratified by the aspect of CRF measured (i.e., $\dot{V}O_{2\max}$, VT, ME, exercise performance). Statistical heterogeneity was assessed using *Q* and *I* ^2^ statistics, with *I* ^2^ values interpreted as negligible (*I* ^2^ = 0%–40%), moderate (*I* ^2^ = 30%–60%), substantial (*I* ^2^ = 50%–90%), or considerable (*I* ^2^ = 75%–100%) (Deeks et al. 2008). We visualized the study‐specific and pooled correlations (95% CIs) using forest plots. We examined sex (male and female) and age (children [mean age < 18 years] and adults [mean age ≥ 18 years]) as potential sources of heterogeneity by performing moderator analyses for random‐effects models and conducted a leave‐one‐out sensitivity analysis. We used an *α* level of 0.05.

### 2.10 Reporting Bias Assessment

When 10 or more studies were included in the meta‐analysis (Higgins et al. 2023), we visually examined the extent of publication bias by funnel plots and formally by Begg and Mazumdar's rank correlation test (Begg and Mazumdar 1994). The influence of publication bias was assessed by Vevea and Woods' (2005) sensitivity analysis for random‐effects models, which corrected the correlation for various publication bias models.

### 2.11 Certainty Assessment

The certainty of the evidence was assessed using a modified Grading of Recommendations, Assessment, Development and Evaluation (GRADE) approach (Schunemann et al. 2013). Certainty assessments were conducted per outcome, with evidence graded as high, moderate, low, or very low. Certainty was based on confidence in the effect estimate and adjusted based on limitations in study design or execution, inconsistency of results, indirectness of evidence, and imprecision. One author (B.G.) assessed the evidence for each outcome and a second author (G.R.T.) verified the assessment for accuracy. Disagreements were resolved through discussion.

## 3 Results

### 3.1 Study Selection

**FIGURE 1:**
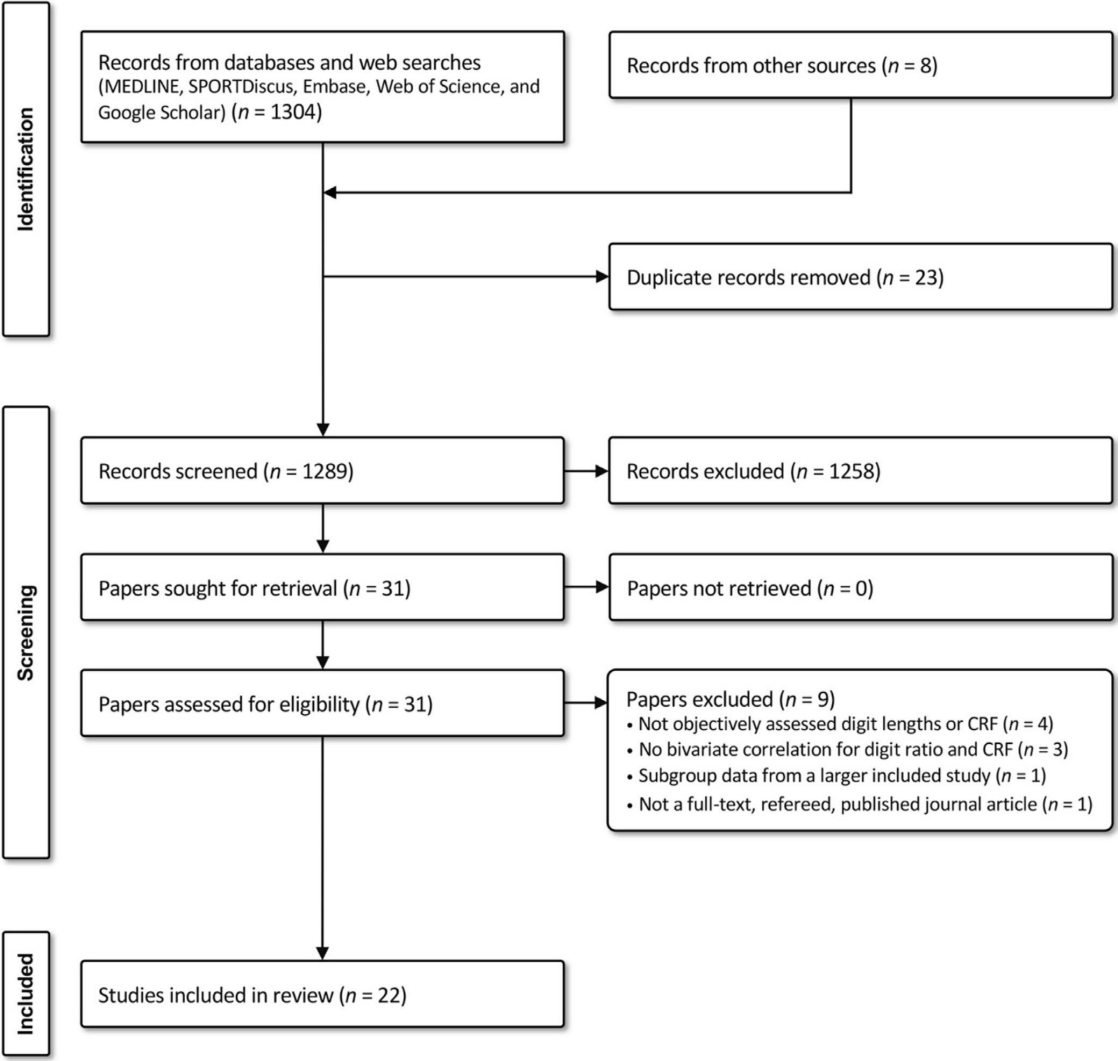
PRISMA flow diagram showing the flow of studies through different phases of the systematic review.

Figure 1 provides a detailed flow diagram of the literature search and screening process, including reasons for full‐text exclusion. A total of 1304 records were identified from online and web searches and 8 records from other sources. After removing 23 duplicates, 1289 records were screened at the title/abstract level, reducing to 31 papers screened in full text. Of these, 9 papers were excluded for not meeting the inclusion criteria, with 22 unique studies included in this meta‐analysis.

### 3.2 Study Characteristics

Table 1 summarizes the descriptive characteristics of the included studies. Studies were published between 2007 and 2024, with almost all (*n* = 21) using non‐probability (i.e., non‐random selection) sampling. There were 5293 individuals from 12 countries classified as having a medium (*n* = 1), high (*n* = 2), or very‐high (*n* = 9) human development index. More than half of participants were male (*n* = 2880 [54%]), ranged in (mean) age from 10.1 to 40.2 years, and comprised school, vocational college, university and military academy students, as well as competitive, elite youth, Olympic and professional athletes. Studies: (a) reported Pearson's (*n* = 20) or Spearman's (*n* = 2) correlation coefficients between 2D:4D and CRF; (b) objectively assessed 2D and 4D lengths either manually (*n* = 16) or electronically (*n* = 6) directly from participants' hands or scans, photographs/copies, or radiographs of participants' hands, and almost always for both hands (*n* = 18); and (c) objectively assessed aspects of CRF using field‐based measures (long‐duration [continuous or interval] running [*n* = 16] or rowing performance [*n* = 1]) or laboratory‐based measures ($\dot{V}O_{2\max}$ [*n* = 5], VT [*n* = 3], or ME [*n* = 1]).

**TABLE 1:** Summary of the 22 included studies (presented in alphabetical order by study author surname).

Author	Country	Sample size (% male)	Mean age (years)	Sampling strategy	Sample description	2D:4D^a^	CRF^b^
Azam et al. (2019)	Malaysia	29 (100%)	10.4	Non‐probability	Kuala Lumpur Football Association (KLFA) academy players	Scans of both hands electronically measured	1609‐m run (s)
Ceylan et al. (2022)	Turkey	60 (50%)	15.0	Non‐probability	Competitive youth basketball players	Both hands manually measured	20‐m shuttle run (laps)
Chen et al. (2022)	China	835 (36%)	19.0^c^	Probability	Vocational college students	Photographs of both hands manually measured	800–1000‐m run (s)
Eklund et al. (2020)	Sweden	20 (0%)	25.9	Non‐probability	Olympic athletes	Right hand manually measured	3000‐m run (min)
Eler (2018)	Turkey	1270 (53%)	10.5^c^	Non‐probability	School children	Photocopies of right hand manually measured	20‐m shuttle run (laps)
Eler et al. (2020)	Turkey	36 (0%)	23.4	Non‐probability	National‐level handball players	Photocopies of both hands manually measured	20‐m shuttle run (laps)
Güler (2018)	Turkey	56 (100%)	16.0	Non‐probability	Competitive youth basketball players	Right hand manually measured	20‐m shuttle run (laps)
Gümüş and Tutkun (2018)	Turkey	23 (100%)	15.1	Non‐probability	Competitive youth football players	Both hands manually measured	20‐m shuttle run (laps)
Hill et al. (2012)	Qatar	41 (100%)	13.9	Non‐probability	Elite youth athletes (football, squash, table tennis, and athletics)	Photocopies of both hands manually measured	$\dot{V}O_{2\max}$ (mL/kg/min)
Holzapfel et al. (2016)	USA	54 (48%)	19.9^c^	Non‐probability	University students (competitive long‐distance runners and sedentary individuals)	Both hands manually measured	$\dot{V}O_{2\max}$ (mL/kg/min) VT (% $\dot{V}O_{2\max}$) ME (mL/kg/min)
Hull et al. (2015)	Australia	69 (0%)	18.8^c^	Non‐probability	National‐level rowers	Photographs of both hands electronically measured	2000‐m rowing (s)
Kociuba et al. (2017)	Poland	167 (0%)	23.7	Non‐probability	Military academy students	Both hands manually measured	12‐min run (m)
Koziel et al. (2017)	Poland	118 (100%)	20.4	Non‐probability	Military academy students	Both hands manually measured	1000‐m run (s)
Lombardo and Otieno (2021)	USA	25 (44%)	14.6^c^	Non‐probability	Competitive youth long‐distance runners	Radiographs of both hands manually measured	$\dot{V}O_{2\text{peak}}$ (mL/kg/min) VT (% $\dot{V}O_{2\text{peak}}$)
Longman et al. (2015)	UK	542 (81%)	31.1^c^	Non‐probability	Competitive long‐distance runners	Photocopies of both hands manually measured	21 098‐m run (s)
Maitra et al. (2021)	India	50 (100%)	40.2	Non‐probability	University students	Both hands manually measured	$\dot{V}O_{2\max}$ (METs)
Manning et al. (2007)	UK	40 (0%)	33.6	Non‐probability	Competitive athletes (athletics)	Photocopies of both hands manually measured	1609‐m run (s)
Nobari et al. (2021)	Iran	24 (100%)	16.1	Non‐probability	Elite youth football players	Scans of both hands electronically measured	30–15 IFT (km/h)
Nobari et al. (2023)	Iran	20 (100%)	13.3	Non‐probability	Elite youth football players	Scans of both hands electronically measured	30–15 IFT (km/h)
Parpa et al. (2024)	Cyprus	133 (100%)	25.2	Non‐probability	Professional football players	Photocopies of both hands manually measured	$\dot{V}O_{2\max}$(mL/kg/min) VT (mL/kg/min)
Ranson et al. (2015)	UK	1654 (53%)	10.1	Non‐probability	School children	Photograph of right hand electronically measured	20‐m shuttle run (laps)
Silva et al. (2022)	Iran	27 (100%)	15.0	Non‐probability	Elite youth football players	Scans of both hands electronically measured	30–15 IFT (km/h)

*Note:* Sampling strategy was coded as probability (i.e., random selection) or non‐probability (i.e., non‐random selection).

Abbreviations: $\dot{V}O_{2\max}$ = maximal oxygen uptake; $\dot{V}O_{2\text{peak}}$ = peak oxygen uptake; 2D:4D = second to fourth digit ratio; 30–15 IFT = 30‐s run (work)–15‐s walk (recovery) intermittent fitness test; CRF = cardiorespiratory fitness; football = soccer; km/h = kilometers per hour; m = meter; ME = mechanical efficiency; METs = metabolic equivalents of task; min = minute; mL/kg/min = milliliters of oxygen per kilogram of body mass per minute; s = second; SD = standard deviation; UK = United Kingdom; USA = United States of America; VT = ventilatory threshold (i.e., the second ventilatory threshold, also called the *respiratory compensation point*).

^a^ Reported for the hand(s) for which bivariate correlations were available.

^b^ Reported as the objectively measured value.

^c^ Mean age estimated as the midpoint of the age range or sample‐weighted mean age.

### 3.3 Risk of Bias in Studies

The risk of bias assessments are summarized in Data S2. The two reviewers agreed on 94% (*n* = 166/176) of JBI appraisal items and demonstrated very high inter‐rater agreement (*κ* = 0.92, 95% CI = 0.87, 0.97). The mean ± SD score was 5.4 ± 0.7 out of a total possible score of 6, which indicated that included studies met almost all critical appraisal criteria and had a low risk of bias. Included studies measured both 2D:4D and CRF in a reliable and valid way and used appropriate statistical analyses. Although most studies sufficiently described the participants and settings, only just over half (*n* = 13) clearly defined the inclusion/exclusion criteria for study participation. Because only studies reporting bivariate correlation coefficients between 2D:4D and aspects of CRF were included, criteria relating to the identification (Item #5) or statistical adjustment (Item #6) of confounding factors were not considered as applicable.

### 3.4 Synthesis of Results

#### 3.4.1 Field‐Based CRF

**FIGURE 2:**
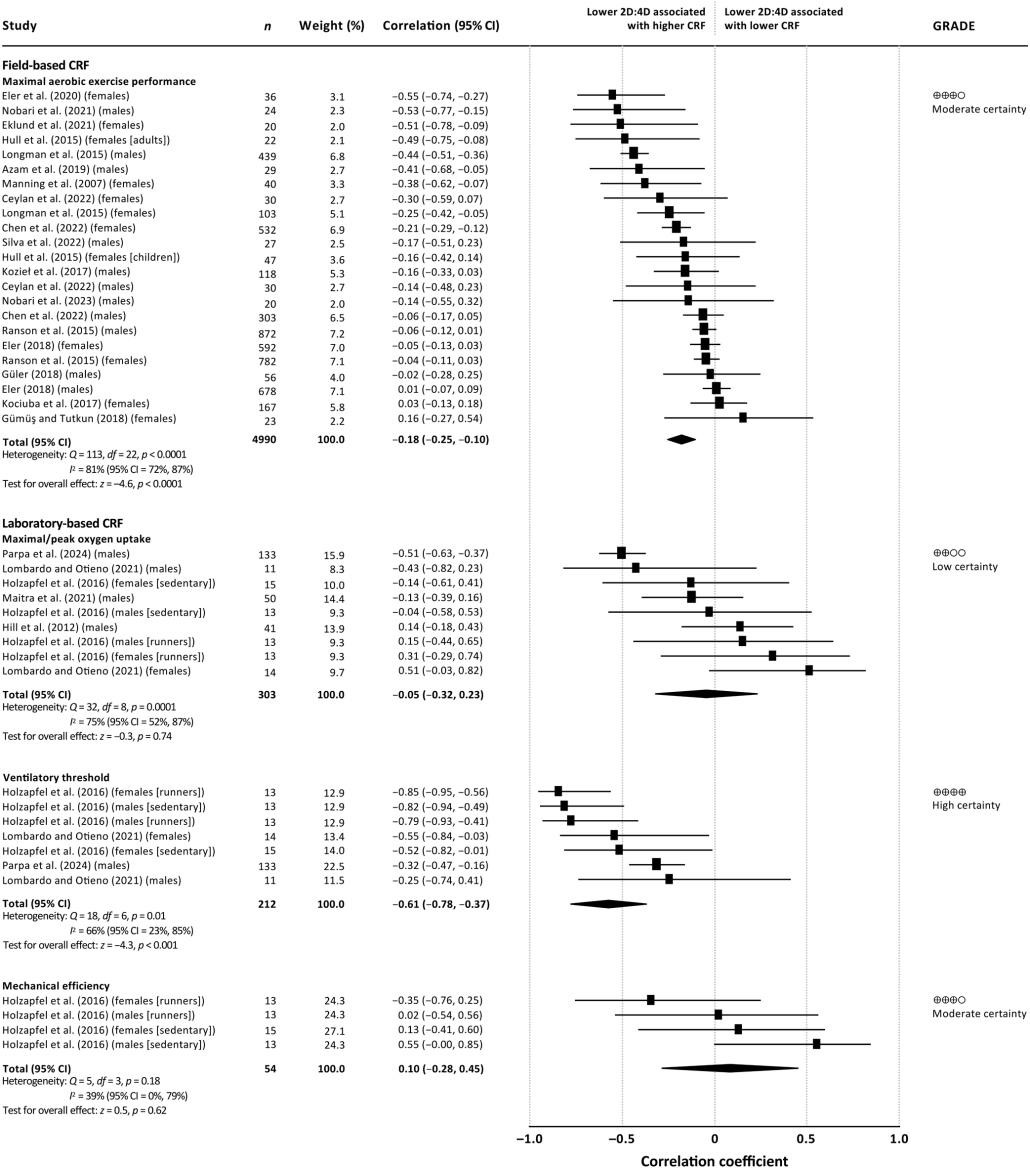
Meta‐analysis of the correlation between digit ratio and aspects of cardiorespiratory fitness. Meta‐analyses were stratified by the aspect of cardiorespiratory fitness into field‐based measures (maximal aerobic exercise performance [long‐duration running/rowing]) and laboratory‐based measures (maximal/peak oxygen uptake, ventilatory threshold, and mechanical efficiency). The squares represent the unique study‐sex‐specific correlations, and the solid horizontal lines represent the corresponding 95% CIs. The center of the diamond represents the pooled correlation, and the lateral tips of the diamond represent the corresponding 95% CI. Negative correlations indicate that individuals with lower digit ratios had higher cardiorespiratory fitness, while positive correlations indicate that individuals with lower digit ratios had lower cardiorespiratory fitness. 2D:4D = second to fourth digit ratio; 95% CI = 95% confidence interval; CRF = cardiorespiratory fitness; df = degrees of freedom; *I* ^2^ = I‐squared statistic; *n* = sample size; *p* = probability value; *Q* = *χ* ^2^ statistic; *z* = *z* statistic.

Figure 2 shows the correlations stratified by CRF measure into field‐based measures (maximal aerobic exercise performance) and laboratory‐based measures ($\dot{V}O_{2\max}$, VT, and ME). Collectively, we found a significant weak negative correlation between 2D:4D and maximal aerobic exercise performance (*r* = −0.18, 95% CI = −0.25, –0.10), indicating that individuals with lower 2D:4Ds performed better physically. There was substantial heterogeneity between studies (*I* ^2^ = 81%, 95% CI = 72%, 87%), with correlations differing by age (*Q* = 5.6, *p* = 0.02; children: *r* = −0.06, 95% CI = −0.11, −0.01; adults: *r* = −0.27, 95% CI = −0.39, −0.14) but not sex (*Q* = 0.4, *p* = 0.53). The removal of each study from the meta‐analysis had a negligible impact (change in *r* ≤ 0.06) (data not shown).

Potential publication bias was indicated by funnel plot analysis (Data S3) and Begg and Mazumdar's test (*τ* = −0.31, *p* = 0.04). Weight‐function sensitivity analysis showed that corrected correlations were minimally affected by moderate and severe publication bias (data not shown).

#### 3.4.2 Laboratory‐Based CRF

We found a significant strong negative correlation between 2D:4D and VT (*r* = −0.61, 95% CI = −0.78, −0.37), indicating that individuals with lower 2D:4Ds had higher VT (Figure 2). The correlations between 2D:4D and both $\dot{V}O_{2\max}$ (*r* = −0.05, 95% CI = −0.32, 0.23) and ME (*r* = 0.10, 95% CI = −0.28, 0.45) were not significant. Between‐study heterogeneity was moderate (ME: *I* ^2^ = 39%, 95% CI = 0%, 79%) to substantial ($\dot{V}O_{2\max}$: *I* ^2^ = 75%, 95% CI = 52%, 87%; VT: *I* ^2^ = 66%, 95% CI = 23%, 85%). There was no significant effect moderation for either sex ($\dot{V}O_{2\max}$: *Q* = 1.9, *p* = 0.16; VT: *Q* = 0.2, *p* = 0.65; ME: *Q* = 1.2, *p* = 0.28) or age ($\dot{V}O_{2\max}$: *Q* = 0.8, *p* = 0.37; VT: *Q* = 0.8, *p* = 0.38; ME: not applicable [only adult data available]). The removal of each study from the meta‐analysis had a negligible impact on the correlation for $\dot{V}O_{2\max}$ (change in *r* ≤ 0.09) and a negligible to small impact on the correlation for VT (change in *r* ≤ 0.27) (data not shown). Note, a leave‐one‐out sensitivity analysis could not be performed for ME because all the correlations came from a single study.

### 3.5 Certainty of Evidence

The certainty of evidence assessments were conducted at the outcome level and are summarized in Data S4 with the accompanying decision rules table. Evidence for $\dot{V}O_{2\max}$ was graded as low certainty due to imprecision and high heterogeneity. Evidence for ME was graded as low certainty due to imprecision and indirectness. Evidence for VT was graded as high certainty (graded down for high heterogeneity but upgraded for a large magnitude of effect). Evidence for maximal aerobic exercise performance was graded as moderate certainty due to high heterogeneity (inconsistency).

## 4 Discussion

This study aimed to systematically review and meta‐analyze studies reporting associations between 2D:4D and objectively assessed aspects of CRF. The findings show that 2D:4D was significantly and negatively associated with VT and exercise performance, but was not significantly associated with $\dot{V}O_{2\max}$ and ME. It appears, therefore, that 2D:4D is a biomarker of some aspects of CRF like exercise tolerance (i.e., VT) and performance, but not others like aerobic capacity and efficiency. These may reflect the long‐term (organizational) benefits of testosterone on aspects of CRF.

A previous meta‐analysis has indicated that 2D:4D is significantly and negatively associated with maximal long‐duration exercise performance (Hönekopp and Schuster 2010). Compared to our meta‐analysis which included only objectively assessed CRF data, Hönekopp and Schuster (2010) pooled and meta‐analyzed both objectively assessed and self/coach‐reported CRF data. Hönekopp and Schuster (2010) also observed stronger relationships between 2D:4D and long‐duration exercise performance requiring high CRF compared to short‐duration exercise performance requiring high muscular fitness. This evidence strengthened the belief that the relationship between 2D:4D and CRF was stronger than that for muscular fitness (Manning et al. 2007; Manning and Hill 2009). Unlike Hönekopp and Schuster (2010) who found that neither sex nor age moderated the association between 2D:4D and sports and athletic performance, we found that age but not sex significantly moderated the association between 2D:4D and aerobic exercise performance (i.e., a more negative association was observed for adults compared to children). By stratifying our meta‐analysis by the aspect of CRF, we examined relationships between 2D:4D and the main physiological mechanisms that describe maximal aerobic exercise performance (Mezzani 2017; Herdy et al. 2016). While we found no significant relationship between 2D:4D and either $\dot{V}O_{2\max}$ or ME, we did find a significant relationship for VT (i.e., the upper intensity limit for prolonged aerobic exercise). This suggests that VT, which accounts for a large proportion of the interindividual variance in aerobic exercise performance (Milani et al. 2006), may explain the observed relationship between 2D:4D and maximal aerobic exercise performance. It is also likely that statistically adjusting for VT will attenuate or eliminate such a relationship. To clarify these potential mechanistic explanations for higher CRF among individuals with lower 2D:4D, future studies should use integrated laboratory‐based measures like cardiopulmonary exercise testing, where multiple aspects of CRF can be concurrently measured. Lower 2D:4D may also be associated with higher CRF through differences in peripheral adaptation or perceived exercise tolerance.

The strongest association between 2D:4D and CRF was found for VT, potentially due to relatively greater variability in VT compared to $\dot{V}O_{2\max}$ and ME among the included samples. Most of the included studies reporting relationships for $\dot{V}O_{2\max}$, VT, and ME comprised small homogenous samples of athletes from the same jurisdiction (e.g., professional football players from Cyprus [Parpa et al. 2024], elite youth athletes from Qatar [Hill et al. 2012], competitive youth runners from the United States [Lombardo and Otieno 2021]). Athlete selection, which represents a “survival of the fittest” whereby strict physical, physiological, skill, and behavioral criteria are imposed, typically results in higher yet less variable levels of fitness among athletes competing at higher levels (Norton and Olds 2001). Reducing the variability in fitness will reduce the magnitude of correlation and may help explain our observed correlational differences. It is possible that the selection pressure for $\dot{V}O_{2\max}$ and ME among the included athlete samples was stronger than that for VT, resulting in more similar athletes and weaker 2D:4D relationships. Future studies with heterogenous samples are required to better understand true relationships between 2D:4D and aspects of CRF and to minimize the effects of range restriction on correlational coefficients. Another possible explanation for the strong negative pooled correlation between 2D:4D and VT may be the relatively larger influence of the study by Holzapfel et al. (2016), which reported bivariate correlations ranging from −0.52 to −0.85 across small samples of 13 to 15 trained and untrained males and females. In contrast, both Lombardo and Otieno (2021) and Parpa et al. (2024) reported weaker correlations between 2D:4D and VT in similarly sized or larger studies, respectively. While Holzapfel et al. (2016) and Lombardo and Otieno (2021) reported non‐significant correlations between 2D:4D and $\dot{V}O_{2\max}$, Parpa et al. (2024) reported an overall significant strong negative correlation. Discrepancies among these studies must be resolved by higher quality future research.

One potential mechanistic explanation for the stronger association between 2D:4D and VT is muscle mass. Theory suggests that individuals with a lower 2D:4D have probably been exposed to higher levels of prenatal testosterone (Manning and Fink 2023; Swift‐Gallant et al. 2020). In turn, prenatal testosterone appears to influence the regulation skeletogenic genes that are responsible for the growth and development of skeletal muscle tissue later in life (Manning et al. 2014; Zheng and Cohn 2011). Higher levels of skeletal muscle tissue should enable individuals to achieve or tolerate higher exercise intensities when expressed as a percentage of $\dot{V}O_{2\max}$ (Mølmen et al. 2024). This increased tolerance to intense exercise may be attributed to improvements in mitochondrial density, capillarization, and arteriovenous oxygen difference, which collectively enhance the body's efficiency in oxygen utilization and aerobic exercise performance. In support of this idea, Manning et al. (2024) found significant moderate‐to‐large positive associations between 2D:4D and the amount of lactate produced during intense incremental exercise, suggesting that lower 2D:4D reflects a greater capacity to tolerate intense exercise.

Psychosocial factors like motivation and the ability to tolerate discomfort are known to affect maximal aerobic exercise performance (Tomkinson et al. 2019). Such psychosocial factors may help explain the significant weak negative correlation we observed between 2D:4D and maximal aerobic exercise performance. Maximal field‐based assessments of CRF are almost always administered to large groups of people who are tested simultaneously, resulting in a competitive physical environment. In competitive or challenging situations, testosterone levels are known to suddenly spike, and the degree to which they spike may be influenced by prenatal testosterone exposure and reflected by the 2D:4D (Cook and Crewther 2012; Crewther et al. 2011, 2022). Individuals with lower 2D:4D are more competitive (aggressive) (Crewther et al. 2015; Kilduff et al. 2013) and have more pronounced spikes in testosterone (Cook and Crewther 2012; Crewther et al. 2011, 2022) during intense exercise compared to individuals with higher 2D:4D. As such, individuals with lower 2D:4D may be more determined and better able to tolerate discomfort (Golby and Meggs 2011), which may translate to better maximal exercise performance. Prenatal testosterone may also influence the motivation to train, leading to more frequent and intense training, which in turn improves discomfort tolerance and maximal exercise performance (Hönekopp et al. 2006). Further investigation into the relationship between 2D:4D and psychosocial factors is warranted to elucidate their contribution to maximal aerobic exercise performance.

### 4.1 Strengths and Limitations

This systematic review represents the most comprehensive synthesis to date of research on the associations between 2D:4D and aspects of CRF. We used strict eligibility criteria and included only studies that reported bivariate correlations between objectively assessed 2D:4D and CRF to reduce heterogeneity between studies and used random‐effects models to incorporate such heterogeneity. As recommended by the PRISMA statement, we also assessed the certainty of the evidence using a modified GRADE approach. Our pooled correlations should be interpreted cautiously because we found moderate to substantial heterogeneity that was neither explained by moderator nor leave‐one‐out sensitivity analyses (except for age which moderated the pooled correlation for exercise performance). While publication bias was indicated for the 2D:4D‐exercise performance meta‐analysis, weight‐function sensitivity analysis indicated that the corresponding correlation was robust and minimally impacted. We neither examined ethnicity as a potential moderator because it was infrequently reported, nor handedness because a previous meta‐analysis (Hönekopp and Schuster 2010) on 2D:4D and sports and athletic performance found that no hand significantly outpredicted the other. Following the recommendation of Aloe and Thompson (2013), we only meta‐analyzed bivariate correlations and neither pooled bivariate correlations nor partial effect sizes in our meta‐analyses, nor ran separate meta‐analyses for bivariate correlations and partial effect sizes. Our meta‐analyses were limited to studies that nearly always used a non‐probability sampling strategy and comprised either small homogenous samples of athletes or larger samples of students who likely had an athletic predisposition. Our findings, therefore, are only generalizable to young athlete and student populations, further highlighting the pressing need to examine relationships between 2D:4D and aspects of CRF using diverse samples.

## 5 Conclusion

We found 2D:4D was significantly and negatively associated with both VT and exercise performance, but was not significantly associated with $\dot{V}O_{2\max}$ and ME. It therefore appears that 2D:4D is a biomarker for some aspects of CRF like exercise tolerance and performance, but not others like aerobic capacity and efficiency. While these data indicate that lower 2D:4D may be linked to an enhanced tolerance for intense exercise, more heterogenous and representative samples are required to better understand true relationships. This review also underscores the need for integrated laboratory‐based measures of CRF to clarify the physiological pathways involved, alongside further exploration of the psychosocial factors that may influence aerobic exercise performance. Nevertheless, this systematic review provides the most comprehensive synthesis of evidence relating 2D:4D and CRF to date.

## Author Contributions

**Bethany Gower:** investigation; methodology; project administration; visualization; writing – original draft preparation; writing – review and editing. **Matthew Russell:** conceptualization; investigation; methodology; project administration; software; writing – review and editing. **Jordan M. Tomkinson:** investigation; writing – original draft preparation; writing – review and editing. **Samantha J. Peterson:** conceptualization; investigation; methodology; supervision; writing review – and editing. **Marilyn G. Klug:** conceptualization; formal analysis; investigation; methodology; supervision; writing – review and editing. **Grant R. Tomkinson:** conceptualization; formal analysis; investigation; methodology; project administration; software; supervision; visualization; writing – original draft preparation; writing – review and editing.

## Ethics Statement

The authors have nothing to report.

## Conflicts of Interest

The authors declare no conflicts of interest.

## Supporting information


**Data S1.** Search strategy for databases.

**Data S2.** Joanna Briggs Institute (JBI) critical appraisal checklist for the 22 included cross‐sectional studies (presented in alphabetical order by study author surname).

**Data S3.** Funnel plot for the correlation between digit ratio and maximal aerobic exercise performance.

**Data S4.** GRADE certainty assessment at the outcome level.

## Data Availability

The analyzed datasets are available from the corresponding authors on reasonable request.
