# Supplementary material for: The Relationship Between Digit Ratio (2D:4D) and Aspects of Cardiorespiratory Fitness: A Systematic Review and Meta‐Analysis
Source: Am J Hum Biol. 2025 Apr 4;37(4):e70040. doi: 10.1002/ajhb.70040 (PMC11969640; doi:10.1002/ajhb.70040)
Supplement: Supplementary file 1 — Data S1. Search strategy for databases. [file AJHB-37-e70040-s003.docx]

**Supplement 1.** Search strategy for databases.

**Table S1a.** Search strategy terms applied in MEDLINE (via Ovid, 1946 to 13 August 2024).

| **#** | **Query** |
| --- | --- |
| 1 | (digit ratio or finger ratio or 2D:4D).mp. [mp=title, book title, abstract, original title, name of substance word, subject heading word, floating sub-heading word, keyword heading word, organism supplementary concept word, protocol supplementary concept word, rare disease supplementary concept word, unique identifier, synonyms, population supplementary concept word, anatomy supplementary concept word] |
| 2 | limit 1 to (full text and humans) |
| 3 | (cardio* or aerobic or exercise or endurance or fitness or oxygen).mp. [mp=title, book title, abstract, original title, name of substance word, subject heading word, floating sub-heading word, keyword heading word, organism supplementary concept word, protocol supplementary concept word, rare disease supplementary concept word, unique identifier, synonyms, population supplementary concept word, anatomy supplementary concept word] |
| 4 | limit 3 to (full text and humans) |
| 5 | 2 and 4 |

**Table S1b.** Search strategy terms applied in SPORTDiscus (via EBSCOhost, 1930­ to 13 August 2024).

| **#** | **Query** |
| --- | --- |
| S1 | digit ratio or finger ratio or 2D:4D |
| S2 | cardio* or aerobic or exercise or endurance or fitness or oxygen |
| S3 | S1 AND S2 |

Limiters: Full Text; Peer Reviewed; Publication Type: Academic Journal; Document Type: Article.

Expanders: Apply related words; Apply equivalent subjects.

Search modes: Boolean/Phrase.

**Table S1c.** Search strategy terms applied in Embase (via Ovid, 1947 to 13 August 2024).

| **#** | **Query** |
| --- | --- |
| 1 | (digit ratio or finger ratio or 2D:4D).mp. [mp=title, abstract, heading word, drug trade name, original title, device manufacturer, drug manufacturer, device trade name, keyword heading word, floating subheading word, candidate term word] |
| 2 | limit 1 to (full text and human) |
| 3 | (cardio* or aerobic or exercise or endurance or fitness or oxygen).mp. [mp=title, abstract, heading word, drug trade name, original title, device manufacturer, drug manufacturer, device trade name, keyword heading word, floating subheading word, candidate term word] |
| 4 | limit 3 to (full text and human) |
| 5 | 2 and 4 |

**Table S1d.** Search strategy terms applied in Web of Science (Core Collection, 1975 to 13 August 2024).

| **#** | **Query** |
| --- | --- |
| 1 | ALL=((digit ratio OR finger ratio OR 2D:4D) AND (cardio* OR aerobic OR exercise OR endurance OR fitness OR oxygen)) |

NOT Document Types: Proceedings Papers or Editorial Materials or Data Papers or Book Chapters or Letters or Meeting Abstracts or Book Reviews or Corrections or Notes or Reprints.

**Table S1e.** Search strategy terms applied in Google Scholar on 13 August 2024 (<https://scholar.google.com>, first 200 results sorted by relevance).

| **#** | **Query** |
| --- | --- |
| 1 | (digit ratio OR finger ratio OR 2D:4D) AND (cardio* OR aerobic OR exercise OR endurance OR fitness OR oxygen) |
