# Supplementary material for: The Relationship Between Digit Ratio (2D:4D) and Aspects of Cardiorespiratory Fitness: A Systematic Review and Meta‐Analysis
Source: Am J Hum Biol. 2025 Apr 4;37(4):e70040. doi: 10.1002/ajhb.70040 (PMC11969640; doi:10.1002/ajhb.70040)
Supplement: Supplementary file 2 — Data S2. Joanna Briggs Institute (JBI) critical appraisal checklist for the 22 included cross‐sectional studies (presented in alphabetical order by study author surname). [file AJHB-37-e70040-s002.docx]

**Supplement 2.** Joanna Briggs Institute (JBI) critical appraisal checklist for the 22 included cross-sectional studies (presented in alphabetical order by study author surname).

| **Author** | **1. Were the criteria for inclusion in the sample clearly defined?** | **2. Were the study subjects and the setting described in detail?** | **3. Was the exposure measured in a valid and reliable way?** | **4. Were objective, standard criteria used for measurement of the condition?** | **5. Were confounding factors identified?** | **6. Were strategies to deal with confounding factors stated?** | **7. Were the outcomes measured in a valid and reliable way?** | **8. Was appropriate statistical analysis used?** |
| --- | --- | --- | --- | --- | --- | --- | --- | --- |
| Azam et al. (2019) | Yes | Yes | Yes | Yes | NA | NA | Yes | Yes |
| Ceylan et al. (2022) | Yes | Yes | Yes | Yes | NA | NA | Yes | Yes |
| Chen et al. (2022) | Yes | Yes | Yes | Yes | NA | NA | Yes | Yes |
| Eklund et al. (2021) | Yes | Yes | Yes | Yes | NA | NA | Yes | Yes |
| Eler (2018) | No | Unclear | Yes | Yes | NA | NA | Yes | Yes |
| Eler et al. (2020) | No | Unclear | Yes | Yes | NA | NA | Yes | Yes |
| Güler (2018) | No | Yes | Yes | Yes | NA | NA | Yes | Yes |
| Gümüş & Tutkun (2018) | No | Yes | Yes | Yes | NA | NA | Yes | Yes |
| Hill et al. (2011) | No | Yes | Yes | Yes | NA | NA | Yes | Yes |
| Holzapfel et al. (2016) | Yes | Yes | Yes | Yes | NA | NA | Yes | Yes |
| Hull et al. (2015) | Yes | Yes | Yes | Yes | NA | NA | Yes | Yes |
| Kociuba et al. (2017) | No | Yes | Yes | Yes | NA | NA | Yes | Yes |
| Koziel et al. (2017) | No | Yes | Yes | Yes | NA | NA | Yes | Yes |
| Lombardo and Otieno (2021) | No | Yes | Yes | Yes | NA | NA | Yes | Yes |
| Longman et al. (2015) | No | Yes | Yes | Yes | NA | NA | Yes | Yes |
| Maitra et al. (2021) | Yes | Unclear | Yes | Yes | NA | NA | Yes | Yes |
| Manning et al. (2007) | Yes | Yes | Yes | Yes | NA | NA | Yes | Yes |
| Nobari et al. (2021) | Yes | Yes | Yes | Yes | NA | NA | Yes | Yes |
| Nobari et al. (2023) | Yes | Yes | Yes | Yes | NA | NA | Yes | Yes |
| Parpa et al. (2024) | Yes | Yes | Yes | Yes | NA | NA | Yes | Yes |
| Ranson et al. (2015) | Yes | Yes | Yes | Yes | NA | NA | Yes | Yes |
| Silva et al. (2022) | Yes | Yes | Yes | Yes | NA | NA | Yes | Yes |

*Notes:* A score of ‘Yes’, ‘No, ‘Unclear’, or ‘Not Applicable’ was given for each appraisal item, with ‘Yes’ and ‘Not Applicable’ answers indicative of a lower risk of bias. The frequency of ‘Yes’ and ‘Not Applicable’ scores were used to indicate the overall risk of bias for each study. Criteria relating to the identification (Item #5) or statistical adjustment (Item #6) of confounding factors were not considered as applicable

*Abbreviations:* NA = Not applicable
