# Supplementary material for: The Relationship Between Digit Ratio (2D:4D) and Aspects of Cardiorespiratory Fitness: A Systematic Review and Meta‐Analysis
Source: Am J Hum Biol. 2025 Apr 4;37(4):e70040. doi: 10.1002/ajhb.70040 (PMC11969640; doi:10.1002/ajhb.70040)
Supplement: Supplementary file 3 — Data S3. Funnel plot for the correlation between digit ratio and maximal aerobic exercise performance. [file AJHB-37-e70040-s001.docx]

**Supplement 3.** Funnel plot for the correlation between digit ratio and maximal aerobic exercise performance.

**
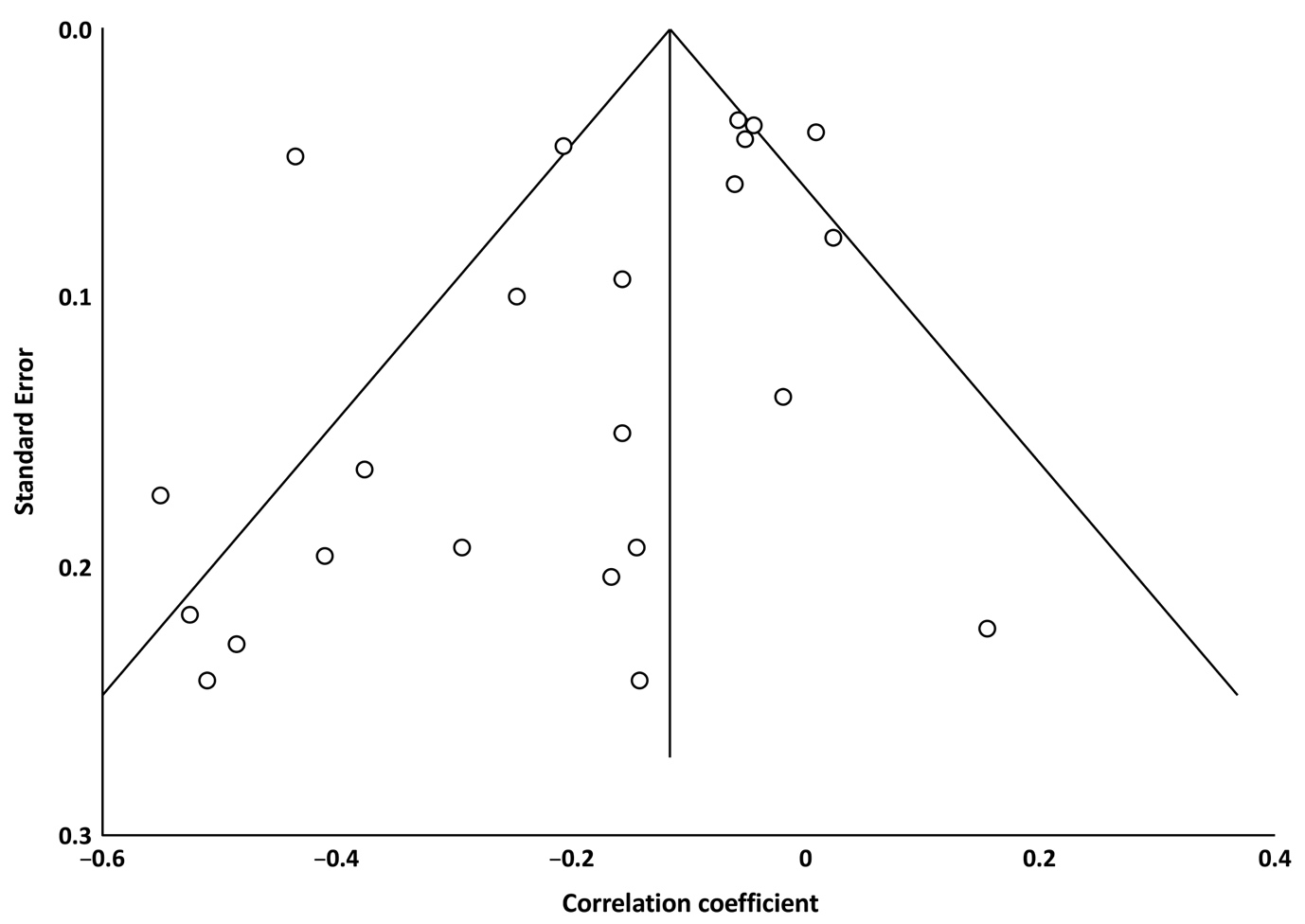
**

**Figure S1.** Funnel plot for the correlation between digit ratio and maximal aerobic exercise performance.

Notes: The circles represent the study-sex-specific correlations for unique participant groups, the dashed vertical line represents the pooled correlation, and the dashed triangular region represents the boundary within which 95% of studies are expected to fall in the absence of both biases and heterogeneity.
