# Supplementary material for: The Relationship Between Digit Ratio (2D:4D) and Aspects of Cardiorespiratory Fitness: A Systematic Review and Meta‐Analysis
Source: Am J Hum Biol. 2025 Apr 4;37(4):e70040. doi: 10.1002/ajhb.70040 (PMC11969640; doi:10.1002/ajhb.70040)
Supplement: Supplementary file 4 — Data S4. GRADE certainty assessment at the outcome level. [file AJHB-37-e70040-s004.docx]

**Supplement 4.** GRADE certainty assessment at the outcome level.

| Aspect of CRF | Risk of bias | Imprecision | Inconsistency | Indirectness | Publication bias | Large magnitude of effect | GRADE |
| --- | --- | --- | --- | --- | --- | --- | --- |
| Maximal aerobic exercise performance | Not serious | Not serious | Serious | Not serious | Not serious | None | ⨁⨁⨁⭘  Moderate |
| Maximal/peak oxygen uptake | Not serious | Serious | Serious | Not serious | Not serious | None | ⨁⨁⭘⭘  Low |
| Ventilatory threshold | Not serious | Not serious | Serious | Not serious | Not serious | Large effect | ⨁⨁⨁⨁  High |
| Mechanical efficiency | Not serious | Serious | Not serious | Serious | Not serious | None | ⨁⨁⭘⭘  Low |

*Abbreviations:* CRF = Cardiorespiratory fitness.

Summary of decision rules: GRADE assessments for certainty of evidence

| **Domain** | **Judgment** | **Scoring** | **Criteria** |
| --- | --- | --- | --- |
| **Risk of Bias*** | No serious RoB | 0 | - Most (>80%) primary studies assessed as having a low risk of bias (e.g., if the study scored “Yes” for most (i.e., ≥4) of the 8 signaling questions). |
|  | Serious RoB | –1 point | - At least one of the primary studies was assessed to be of high risk of bias, but the relative contribution of each study to the overall results will be considered for assessing the overall level of bias (e.g., if the studies scored “Seek more info” if we were unclear about several (≥3) signaling questions). |
| **Imprecision** | No serious imprecision | 0 | - The 95% CIs for the pooled correlations do not include 0. |
|  | Serious imprecision | –1 point | - The 95% CIs for the pooled correlations include 0 |
| **Inconsistency** | No serious inconsistency | 0 | - Statistical tests for heterogeneity in the meta-analysis are not significant and the I^2^ was negligible to moderate (i.e., <50%). |
|  | Serious inconsistency | –1 point | - Heterogeneity in the meta-analysis substantial (i.e., ≥50%) |
| **Indirectness** | No serious indirectness | 0 | - There was good representation across the primary studies underpinning the aspect of CRF (e.g., variety of populations, sex distribution). - The tests used to assess health outcome/s all used objective measurement (e.g., cardiopulmonary exercise testing). |
|  | Serious indirectness | –1 point | The studies underpinning the aspect of CRF were limited by having one of the following:   - Limited representation of primary studies with consideration for their relative contribution to the estimate. - Considerable inclusion of participants <18 years or only one sex captured in the studies or only one ethnicity captured in the studies. |
| **Publication Bias** | No serious publication bias | 0 | - There is no suspected evidence of publication bias as reported in the systematic review based on Begg and Mazumdar’s test, visual inspection of funnel plots, and/or weight-function sensitivity analysis. - There were an insufficient number of included studies to assess publication bias (<10 studies). |
|  | Serious publication bias | –1 point | - There is evidence of publication bias arising from asymmetrical rather than symmetrical funnel plots, or if statistical tests of asymmetry were positive. |

CI – confidence interval, CRF – cardiorespiratory fitness, RoB – risk of bias

*Risk of bias will be based on the quality assessment of the primary studies included in the systematic reviews. If a RoB assessment was not provided within the systematic review, a de novo RoB assessment was conducted using the Joanna Briggs Institute (JBI) critical appraisal checklist for analytical cross-sectional studies tool.

- The quality of the evidence was upgraded if there was evidence of a large magnitude of effect from meta-analyses with sufficiently narrow confidence intervals. Large effect was defined as a magnitude of effect value > 0.2.
- All outcomes started at a rating of ‘high’ certainty per PERSIST guidelines (Ardern, C.L., Büttner, F., Andrade, R., Weir, A., Ashe, M.C., Holden, S., Impellizzeri, F.M., Delahunt, E., Dijkstra, H.P., Mathieson, S. and Rathleff, M.S., 2022. Implementing the 27 PRISMA 2020 Statement items for systematic reviews in the sport and exercise medicine, musculoskeletal rehabilitation and sports science fields: the PERSiST (implementing Prisma in Exercise, Rehabilitation, Sport medicine and SporTs science) guidance. British journal of sports medicine, 56(4), pp.175-195.)
- The quality of the evidence **per each outcome** can be interpreted as follows (6):

**High:** we are confident that the true magnitude of effect between 2D:4D and the aspect of CRF lies close to the effect estimated in the meta-analysis and further research is unlikely to change our confidence in the magnitude of effect.

**Moderate:** we are moderately confident that the true magnitude of effect between 2D:4D and the aspect of CRF is likely to be close to the effect estimated in the meta-analysis, but there is a possibility that it is substantially different; further research is likely to have an important impact on the confidence in the direction of association and may change the direction of association.

**Low:** we have limited confidence; the true magnitude of effect between 2D:4D and the aspect of CRF may be substantially different from the estimate.

**Very low:** we have very little confidence; the true magnitude of effect between 2D:4D and the aspect of CRF may be substantially different from the estimate.
